# Supplementary material for: Malaria prevalence and use of control measures in an area with persistent transmission in Senegal
Source: PLoS One. 2024 May 16;19(5):e0303794. doi: 10.1371/journal.pone.0303794 (PMC11098374; doi:10.1371/journal.pone.0303794)
Supplement: S1 File — (DOCX) [file pone.0303794.s002.docx]

Date de l’enquête: ___/___/_______/

Nom de l’enquêteur: ________________________________________

| **COORDONNEES GPS DE LA CONCESSION DU PARTICIPANT** | | | |
| --- | --- | --- | --- |
|  | Longitude (à automatiser) |  | |
|  | Latitude (à automatiser) |  | |
|  | **IDENTIFICATION DU PARTICIPANT** | | |
|  | District sanitaire |  | |
|  | Poste de santé | 1. Diakhaling 2. Khossanto 3. Mamakhono 4. Sambrambougou | |
|  | Nom du village (s’affiche en fonction du poste chosi)  Si poste=Diakhaling, village: | 1. Tourokhoto 2. Kobokhoto 3. Diegoun 4. Diakhaling 5. Dindifa | |
|  | Si poste=Mamakhono, village: | 1. Bambaraya 2. Bambarayading 3. Mamakhono | |
|  | Si poste=Khossanto, village: | 1. Khossanto 2. Madina Berola 3. Lefakho 4. Niamahya 5. Mandankholing | |
|  | Si poste=Sambrambougou, village: | 1. Sambranbougou 2. Douta | |
|  | Code du participant (doit être alphanumérique) | I____I____I____I____I____I____I (copier le numero sur l’étiquette) | |
|  | Ethnie | Sarakole  Wolof  Pular  Bassari  Autre, spécifiez ________________ | |
|  | Age | I____I____I____I mois. Indiquer l’âge en mois si le sujet est âgé de moins de 1 an  I____I____I années (indiquer en année pour les autres) | |
|  | Date de naissance | I____I____I____I____I____I____I____I____\| indiquer la date de naissance si l’âge n’est pas disponible) | |
|  | Sexe | 1. \|___\| Masculin 2. \|___\|. Féminin | |
|  | Occupation (indiquez à partir de 5 ans) | 1. Elève 2. Berger 3. Orpailleur 4. Enseignant 5. Cultivateur 6. Vendeur/Commerçant 7. Chauffeur de Taxi 8. Autre, spécifiez ___________________ | |
|  | Niveau d’éducation | 1. Ecole coranique 2. Primaire 3. Secondaire 4. Universitaire 5. Aucune 6. Autre, spécifiez ______________________ | |
| 1. **Identification du chef de ménage du participant (SI LE PARTICIPANT n’est pas le chef de menage)** | | | |
|  | Initiales du chef de ménage |  | |
|  | Niveau d’éducation | 1. Ecole coranique 2. Niveau primaire 3. Niveau secondaire 4. Niveau universitaire 5. Aucune 6. Autre, specifiez_______________________ | |
|  | Occupation | Berger  Orpailleur  Enseignant  Cultivateur  Vendeur/Commerçant  Chauffeur de Taxi  Autres, spécifiez _________________________________________________ | |
|  | Sexe | 1. \|___\| Masculin 2. \|___\|. Féminin | |
| 1. **Caractéristiques du ménage** | | | |
|  | Nombre de personnes dans le ménage | \|___\| (indiquez le nombre) | |
|  | Type de toit | 1. Tôle 2. Ciment/Beton 3. Paille/chaume 4. Carton 5. Planches 6. Autres, spécifiez | |
|  | Type de mur | 1. Ciment 2. Terre battue/boue 3. Bois/planches 4. Bambou 5. Autres : _________________________ | |
|  | Type de sol | 1. Ciment 2. Carreaux 3. Sable 4. Planches de bois 5. Autres : _______________________ | |
|  | Source d’eau | 1. Robinet, 2. Puits dans la maison, 3. Puits public 4. Camion citerne 5. Eau de pluie 6. Autres: _______________________ | |
|  | Type de toilette | 1. Toilette personnelle 2. Toilette commune 3. Latrine personnelle 4. Latrine commune 5. Absence de toilette 6. Autre, spécifiez___________________________ | |
|  | Type de combustible de cuisson | 1. Bois de chauffage 2. Gaz 3. Électricité 4. Pétrole 5. Charbon 6. Autre, spécifiez_________________________ | |
|  | Possession de biens | | |
|  | Radio | \|____\| Oui/non | |
|  | Television | \|____\| Oui/non | |
|  | Velo | \|____\| Oui/non | |
|  | Scooter | \|____\| Oui/non | |
|  | Voiture | \|____\| Oui/non | |
|  | Refrigérateur | \|____\| Oui/non | |
|  | Ventilateur | \|____\| Oui/non | |
|  | Telephone portable | \|____\| Oui/non | |
|  | Charrettes | \|____\| Oui/non | |
|  | Bétails | \|____\| Oui/non | |
|  | **Histoire de voyage** | | |
|  | Au cours des quatre dernières semaines, avez-vous voyagé en dehors du village ? | | 1. Oui 2. Non |
|  | Si oui, combien de jours êtes-vous restés en dehors du village ? | | **\|_____\|____\| jours** |
|  | Avez-vous utilisé la moustiquaire quand vous étiez en voyage ? | | 1. Oui 2. Non |
|  | **Prévention du paludisme** | | |
|  | Possédez vous des moustiquaires dans le ménage? | 1. Oui 2. Non | |
|  | Si, oui combien | \|  \| \| --- \|   (Indiquez le nombre) | |
|  | Dormez vous sous moustiquaire? | 1. Oui 2. Non | |
|  | Si, oui à quelle saison | 1. 1, Saison pluvieuse 2. 2, Saison sèche 3. 3, Toutes les saisons 4. 4, NSP | |
|  | A quelle fréquence, dormez vous sous moustiquaire? | 1. Toutes les nuits 2. 3 à 6 fois par semaine 3. moins de 3 fois (0, 1, 2 fois) par semaine | |
|  | Avez- vous dormi sous moustiquaire la nuit dernière? | 1. Oui 2. Non | |
|  | Si non, pourquoi | 1. Très chaud 2. N’aime pas l’odeur 3. Je me sens «enfermé » 4. Pas de paludisme actuellement 5. Pas de moustiques 6. Moustiquaire trop veille et déchirée 7. Autre, spécifiez _____________________ | |
|  | Utilisez-vous d’autres moyens de prevention? | 1. Oui 2. Non | |
|  | Si, oui indiquez les autres moyens que vous utilisez | 1. Serpentin fumigène 2. Insecticide (ex. yotox) 3. Désherbage 4. Évacuation des eaux usées 5. Port d'habits longs 6. Autre, spécifiez ______________________ | |
|  | OBSERVATION - INSPECTION PHYSIQUE DE LA MOUSTIQUAIRE UTILISÉE LA NUIT DERNIÈRE | | |
|  | Comment se présente la moustiquaire? | 1. Attaché et bordé 2. Attaché et suspendu 3. Disponible mais pas attaché 4. Non vu | |
|  | La moustiquaire a t-elle des trous? | 1. Oui 2. Non | |
|  | Si oui, décrire les trous |  | |
|  | **a**. Trous de taille 1 (*Moins que la taille d’un doigt (0.5 – 2 cm)* | **Emplacement** | |
|  |  | 1. Toit | |
|  |  | 1. Zone supérieure | |
|  |  | 1. Zone inférieure | |
|  |  | 1. Zone de couture | |
|  | **b.** Trous de taille 2 (*Aussi large qu’un doigt, plus grand que le trou de taille 1 (2 -10 cm)* | 1. Toit | |
|  |  | 1. Zone supérieure | |
|  |  | 1. Zone inférieure | |
|  |  | 1. Zone de couture | |
|  | **c.** Trous de taille 3 (*Plus large que le trou de taille 2 (10 – 25 cm)* | 1. Toit | |
|  |  | 1. Zone supérieure | |
|  |  | 1. Zone inférieure | |
|  |  | 1. Zone de couture | |
|  | **d.** Trous de taille 4  *Plus large que le trou de taille 3 (>25 cm)* | 1. Toit | |
|  |  | 1. Zone supérieure | |
|  |  | 1. Zone inférieure | |
|  |  | 1. Zone de couture | |
|  | INFORMATIONS SUR LA CPS (POUR LES SUJETS DE 6 MOIS À 10 ANS) | | |
|  | Avez reçu la CPS cette année? | Oui  Non | |
|  | Si oui, indiquer le nombre de doses (se réferer à la carte CPS) | \| Mois \| Traité (Oui/Non) \| Nombre de doses \| \| --- \| --- \| --- \| \| 1^er^ passage (juin) \|  \|  \| \| 2è passage (juillet) \|  \|  \| \| 3è passage (août) \|  \|  \| \| 4è passage (septembre) \|  \|  \| | |
|  |  |  | |
| 1. **Examens réalisés (demandez au technicien)** | | | |
|  | La Goutte épaisse a-t-elle été réalisée? | 1. Oui 2. Non | |
|  | Le frottis sanguin a t-il été réalisé? | 1. Oui 2. Non | |
|  | Le prélèvement sur papier filtre a t-il été réalisé? | 1. Oui 2. Non | |
|  | Le prélèvement sur tube Eppendorf a t-il-été réalisé? | 1. Oui 2. Non | |
|  | Le taux d’hémoglobine a -t-il été mesuré? | 1. Oui 2. Non | |
|  | Si oui, indiquez la valeur du taux d’hémoglobine | \|____\|____\|. \|____\| g/dl | |
|  | Temperature | \|____\|____\|. \|____\| degrés Celcius | |
