# Supplementary material for: Malaria prevalence and use of control measures in an area with persistent transmission in Senegal
Source: PLoS One. 2024 May 16;19(5):e0303794. doi: 10.1371/journal.pone.0303794 (PMC11098374; doi:10.1371/journal.pone.0303794)
Supplement: S2 File — (DOCX) [file pone.0303794.s003.docx]

QUESTIONNAIRE FOR THE PREVALENCE STUDY

Survey date: ___/___/_______/

Name of enumerator: ________________________________________

| **GPS COORDINATES OF PARTICIPANT'S HOUSE** | | | |
| --- | --- | --- | --- |
|  | Longitude |  | |
|  | Latitude |  | |
|  | **IDENTIFICATION OF THE PARTICIPANT** | | |
|  | Health district |  | |
|  | Health post | Diakhaling  Khossanto  Mamakhono  Sambrambougou | |
|  | Village name (displayed according to the health post selected)  If health post=Diakhaling | 1, Tourokhoto  2, Kobokhoto  3, Diegoun  4, Diakhaling  5, Dindifa | |
|  | If health post=Mamakhono | 1, Bambaraya  2, Bambarayading  3, Mamakhono | |
|  | If health post=Khossanto | 1, Khossanto  2, Madina Berola  3, Lefakho  4, Niamahya  5, Mandankholing | |
|  | If health post=Sambrambougou | 1, Sambranbougou  2, Douta | |
|  | Participant ID (must be alphanumeric) | I____I____I____I____I____I (copy number from label) | |
|  | Ethnicity | 1. Sarakole 2. Wolof 3. Pular 4. Bassari 5. Other, please specify | |
|  | Age | I____I____I____I months. Indicate age in months if the subject is less than 1 year old.  I____I____I years (indicate in years for others) | |
|  | Date of birth | I____I____I____I____I____I____I____I____\| indicate date of birth if age not available) | |
|  | Gender | 1. \|___\| Male 2. \|___\|. Female | |
|  | Occupation (indicate from age >=5) | 1. Student 2. Shepherd 3. Gold digger 4. Teacher 5. Farmer 6. Vendor/trader 7. Taxi Driver 8. Other, specify | |
|  | Education level | 1. Koranic School 2. Primary 3. Secondary 4. University 5. None 6. Other, specify | |
| 1. **Identification of the participant's head of household (IF THE PARTICIPANT IS NOT THE HEAD OF HOUSEHOLD)** | | | |
|  | Initials of the household head |  | |
|  | Education level | 1. Koranic school 2. Primary 3. Secondary 4. University 5. None 6. Other, specify | |
|  | Occupation | 1. Shepherd 2. Gold digger 3. Teacher 4. Farmer 5. Seller/Dealer 6. Taxi Driver 7. Other, specify __________________________________________ | |
|  | Gender | 1. \|___\| Male 2. \|___\|. Female | |
| 1. **Household characteristics** | | | |
|  | Number of people in household | \|___\| | |
|  | Roof type | 1. Sheet metal 2. Cement/Beton 3. Straw/stubble 4. Cardboard 5. Plank 6. Other, specify | |
|  | Wall type | 1. Cement 2. Rammed earth/mud 3. Wood/boards 4. Bamboo 5. Other, specify _________________________ | |
|  | Soil type | 1. Cement 2. Tiles 3. Sand 4. Wooden boards 5. Other, specify _______________________ | |
|  | Water source | 1. Tap, 2. Well in the house, 3. Public well 4. Tank truck 5. Rainwater 6. Other, specify______________________ | |
|  | Toilet type | 1. Personal toilet 2. Shared toilet 3. Personal latrine 4. Shared latrine 5. No toilet 6. Other, specify___________________________ | |
|  | Type of cooking fuel | 1. Firewood 2. Gas 3. Electricity 4. Kerosene 5. Coal 6. Other, specify_________________________ | |
|  | Possession of goods | | |
|  | Radio | \|____\| Yes/No | |
|  | Television | \|____\| Yes/No | |
|  | Bike | \|____\| Yes/No | |
|  | Scooter | \|____\| Yes/No | |
|  | Car | \|____\| Yes/No | |
|  | Refrigerator | \|____\| Yes/No | |
|  | Fan | \|____\| Yes/No | |
|  | Cell phone | \|____\| Yes/No | |
|  | Carts | \|____\| Yes/No | |
|  | Cattle | \|____\| Yes/No | |
|  | **Travel history** | | |
|  | Have you travelled outside the village in the last four weeks? | | 1. Yes 2. No |
|  | If so, how many days did you stay outside the village? | | **\|_____\|____\| days** |
|  | Did you use the mosquito net when you were travelling? | | 1. Yes 2. No |
|  | **Malaria prevention** | | |
|  | Do you have bed nets in the household? | 1. Yes 2. No | |
|  | If so, how much | \|____\| (indicate the number) | |
|  | Do you sleep under a bed net? | 1. Yes 2. No | |
|  | If yes, in which season | 1. Rainy season 2. Dry season 3. All seasons 4. Don’t Know | |
|  | How often do you sleep under a bed net? | 1. Every night 2. 3 to 6 times a week 3. Less than 3 times (0, 1, 2) per week | |
|  | Did you sleep under a bed net last night? | 1. Yes 2. No | |
|  | If no, why not | 1. Very hot 2. Don't like the smell 3. I feel "locked in » 4. No malaria at present 5. No mosquitoes 6. Mosquito net too old and torn 7. Other, specify _____________________ | |
|  | Do you use other means of prevention? | 1. Yes 2. No | |
|  | If yes, please indicate the other means you use | 1. Smoke coil 2. Insecticide (e.g. yotox) 3. Weeding 4. Waste water disposal 5. Wearing long clothes 6. Other, specify_____________________ | |
|  | OBSERVATION - PHYSICAL INSPECTION OF THE BED NET USED THE NIGHT BEFORE THE SURVEY | | |
|  | How does the bed net look? | 1. Tied and lined 2. Attached and suspended 3. Available but not attached 4. Not seen | |
|  | Does the bed et have holes? | 1. Yes 2. No | |
|  | If so, describe the holes |  | |
|  | **a**. Hole size 1 (*Less than the size of a finger (0.5 - 2 cm*) | **Location of the holes** | |
|  |  | 1. Roof | |
|  |  | 1. Upper zone | |
|  |  | 1. Lower zone | |
|  |  | 1. Sewing zone | |
|  | **b.** Size 2 holes (*as wide as a finger, larger than size 1 hole (2 -10 cm*)) | 1. Roof | |
|  |  | 1. Upper zone | |
|  |  | 1. Lower zone | |
|  |  | 1. Sewing zone | |
|  | **c.** Hole size 3 (*wider than hole size 2 (10 - 25 cm*)) | 1. Roof | |
|  |  | 1. Upper zone | |
|  |  | 1. Lower zone | |
|  |  | 1. Sewing zone | |
|  | **d.** Size 4 holes  *Wider than size 3 hole (>25 cm)* | 1. Roof | |
|  |  | 1. Upper zone | |
|  |  | 1. Lower zone | |
|  |  | 1. Sewing zone | |
|  | INFORMATION ON CPS (FOR SUBJECTS AGED 6 MONTHS TO 10 YEARS) | | |
|  | Did you receive SMC this year? | 1. Yes 2. No | |
|  | If yes, indicate number of doses (refer to CPS card) | \| Month \| recieved (Yes/No) \| Number of doses \| \| --- \| --- \| --- \| \| 1^st^ round (June) \|  \|  \| \| 2^nd^ round (July) \|  \|  \| \| 3^rd^ round (August) \|  \|  \| \| 4^th^ round (September) \|  \|  \| | |
|  |  |  | |
| 1. **Tests performed (ask the technician)** | | | |
|  | Has the thick blood smear been completed? | 1. Yes 2. No | |
|  | Has the thin blood smear been taken? | 1. Yes 2. No | |
|  | Was the filter paper sample taken? | 1. Yes 2. No | |
|  | Was the Eppendorf tube sample taken? | 1. Yes 2. No | |
|  | Has the hemoglobin level been measured? | 1. Yes 2. No | |
|  | If yes, please indicate hemoglobin value | \|____\|____\|. \|____\| g/dl | |
|  | Temperature | \|____\|____\|. \|____\| Celsius degres | |
