# Supplementary material for: Malaria prevalence and use of control measures in an area with persistent transmission in Senegal
Source: PLoS One. 2024 May 16;19(5):e0303794. doi: 10.1371/journal.pone.0303794 (PMC11098374; doi:10.1371/journal.pone.0303794)
Supplement: Attachment — Submitted filename: Response to Reviewers..docx [file pone.0303794.s005.docx]

To PLOS ONE editor

**Subject**: Response to Reviewers

**Ref**: PONE-D-23-41641

Malaria prevalence and use of control measures in an area with persistent transmission in Senegal

Dear Editor;

On behalf of the co-authors, I would like to thank the editor and the reviewers for the comments and submit below the point-by-point responses to the concerns raised during the review process:

1. Please ensure that your manuscript meets PLOS ONE's style requirements, including those for file naming. The PLOS ONE style templates can be found at
   https://journals.plos.org/plosone/s/file?id=wjVg/PLOSOne_formatting_sample_main_body.pdf and 
   <https://journals.plos.org/plosone/s/file?id=ba62/PLOSOne_formatting_sample_title_authors_affiliations.pdf>"

Authors’ reply: The manuscript has been revised in accordance with PLOS ONE’s style requirements, including those for file naming

2. Thank you for stating in your Funding Statement:

“The study was supported by the Senegalese National Malaria Control Program, through an agreement with the Department of Parasitology and Mycology of University Cheikh Anta Diop of Dakar (# funding number: NFM 2-SEN-M-PNLP 2018 - 2019 -2020). The funder had no role in the study design, data collection, and analysis, decision to publish, or preparation of the manuscript”

Please provide an amended statement that declares *all* the funding or sources of support (whether external or internal to your organization) received during this study, as detailed online in our guide for authors at http://journals.plos.org/plosone/s/submit-now.  Please also include the statement “There was no additional external funding received for this study.” in your updated Funding Statement.

Please include your amended Funding Statement within your cover letter. We will change the online submission form on your behalf.

Authors’ reply: The amended statements (see below) have been included in the cover letter:

The study was supported by the Senegalese National Malaria Control Program, through an agreement with the Department of Parasitology and Mycology of University Cheikh Anta Diop of Dakar (# funding number: NFM 2-SEN-M-PNLP 2018 - 2019 -2020). There was no additional external funding received for this study. The funders had no role in study design, data collection and analysis, decision to publish, or preparation of the manuscript.

3. We note that the grant information you provided in the ‘Funding Information’ and ‘Financial Disclosure’ sections do not match.

When you resubmit, please ensure that you provide the correct grant numbers for the awards you received for your study in the ‘Funding Information’ section.

Authors’reply: The correct grant number is (NFM 2-SEN-M-PNLP 2018 - 2019 -2020).

4. Please include a separate caption for each figure in your manuscript.

Authors’reply: The manuscript has been revised to reflect separate caption for each figure

5. We note that Figure 1 in your submission contain [map/satellite] images which may be copyrighted. All PLOS content is published under the Creative Commons Attribution License (CC BY 4.0), which means that the manuscript, images, and Supporting Information files will be freely available online, and any third party is permitted to access, download, copy, distribute, and use these materials in any way, even commercially, with proper attribution. For these reasons, we cannot publish previously copyrighted maps or satellite images created using proprietary data, such as Google software (Google Maps, Street View, and Earth). For more information, see our copyright guidelines: http://journals.plos.org/plosone/s/licenses-and-copyright.

We require you to either (1) present written permission from the copyright holder to publish these figures specifically under the CC BY 4.0 license, or (2) remove the figures from your submission:

 a. You may seek permission from the original copyright holder of Figure 1 to publish the content specifically under the CC BY 4.0 license.

We recommend that you contact the original copyright holder with the Content Permission Form (http://journals.plos.org/plosone/s/file?id=7c09/content-permission-form.pdf) and the following text:

“I request permission for the open-access journal PLOS ONE to publish XXX under the Creative Commons Attribution License (CCAL) CC BY 4.0 (http://creativecommons.org/licenses/by/4.0/). Please be aware that this license allows unrestricted use and distribution, even commercially, by third parties. Please reply and provide explicit written permission to publish XXX under a CC BY license and complete the attached form.”

Please upload the completed Content Permission Form or other proof of granted permissions as an "Other" file with your submission.

In the figure caption of the copyrighted figure, please include the following text: “Reprinted from [ref] under a CC BY license, with permission from [name of publisher], original copyright [original copyright year].”

b. If you are unable to obtain permission from the original copyright holder to publish these figures under the CC BY 4.0 license or if the copyright holder’s requirements are incompatible with the CC BY 4.0 license, please either i) remove the figure or ii) supply a replacement figure that complies with the CC BY 4.0 license. Please check copyright information on all replacement figures and update the figure caption with source information. If applicable, please specify in the figure caption text when a figure is similar but not identical to the original image and is therefore for illustrative purposes only.

Authors’reply: Fig 1 illustrating the study site was created by one of the co-authors (Mr Libasse Sarr) for this article, and was not previously published anywhere. A permission to publish the figure under the Creative Commons Attribution License (CCAL) CC BY 4.0 is requested from him and the Content Permission Form is submitted along with the revised version of the manuscript.

6. Additional Editor Comments (if provided):

The paper is very relevant and timely. The study is well designed and easily explained. The manuscript is well written in a very simple and accessible language.

Results are well presented with their respective public health implications. Lastly key recommendations are given to strenghten malaria surveillance in all age groupes and support national effort towards malaria elimination. However, Saraya being a high transmission area where SMC is implemented since almost 10 years, we cannot understand that SMC data was not collected among children under 10 years. The authors did not give any explanation on the absence of SMC data during data collection and analysis. The absence of SMC data is a major limit that needs to be adressed by the authors.

Authors’reply: The study collected data on SMC coverage among children under 10 years. These data are presented in the revised version as follow:

According to the report of caregivers/mothers, a proportion of sixty-eight percent (68.8%) of children aged 6 months to 10 years (n=839) received at least one dose of SMC; full coverage of SMC (3 days of treatment for all four rounds) was evaluated at 46.8% (see Table 1, page 13)

**Reviewer #1**:

1. This study was conducted in a high malaria transmission area of Senegal, the district of Saraya where seasonal malaria chemoprevention (SMC) is implemented for children under 10 years of age. In Saraya district’s, under 10 children receive monthly a curative dose of antimalarial during the malaria transmission season from June to October.

Why don’t the authors include the SMC coverage in their data collection?

I think that conducting a malaria prevalence study in an area like Saraya should take into account the coverage rate for a better interpretation of the results. I recommend that authors include the CMS coverage rate in their data collection.

Authors’reply : The study collected data on SMC coverage among children under 10 years. These data are presented in the revised version as follow:

According to the report of caregivers/mothers, a proportion of sixty-eight percent (68.8%) of children aged 6 months to 10 years (n=839) received at least one dose of SMC; full coverage of SMC (3 days of treatment for all four rounds) was evaluated at 46.8% (see Table 1, page 13)

2. Is the low prevalence of asymptomatic and symptomatic malaria in children under 10 not due to SMC?

Authors’reply: Though SMC data was collected in this study, we did not find any evidence of SMC effect on symptomatic or asymptomatic *P. falciparum* malaria due to lack of power. Indeed, assessing SMC effect on symptomatic and asymptomatic infections, will require subgroup analysis with restrictions on SMC targets who were found with *P. falciparum* infections either symptomatic or asymptomatic (N=198). This prevalence study was designed to assess malaria prevalence at all age group but was not powered to detect an effect of SMC on symptomatic or asymptomatic malaria.

3. Corrections are also needed for Plasmodium species. P. falciparum instead of P. Falciparum

Authors’reply: Plasmodium species “P. falciparum” has been harmonized throughout the manuscript

**Reviewer #2**:

1. Abstract:

Page 3 Line 50-51 & 56-57. There are discrepancies between the statement on malaria by age group.

Line 50-51. “There was no statistical difference in malaria infection by age group.”

Line 56-57. “Participants aged 5-9 years were more likely to have malaria infection compared to under five children (aOR=1.40, 95% CI:1.02-1.91).”

Authors’reply: The authors agreed with the reviewer.

Line 50-51 refer to univariate analysis and this has showed a significant effect of age on malaria prevalence (specifically between children aged 5-10 years old and adults aged ≥20 years old). To avoid confusion with the multivariate analysis, the manuscript is revised and the sentence in Line 50-51 is removed.

2. Introduction:

Page 4. Line 74-78. It was observed that you used different indices for malaria mortality, which did not allow for an easy comparison of the trend in malaria mortality between 2020 and 2022. You are advised to use the same metric for comparison to ensure objectivity.

Authors’reply: This section has been revised in the manuscript. “Mortality rate” is used to compare malaria mortality between 2020-2022 (Please, see Line 78-80)

3. Method:

Page 6. Line 113. You will need to be explicit on inclusion and exclusion criteria as individuals on antimalaria may be asymptomatic at the time of the recruitment and should have been excluded as they may still harbour the parasite.

Authors’reply: The manuscript has been revised to take into account the reviewer’s comment; inclusion and exclusion criteria considered during the recruitment are added to the revised manuscript (Please, see Line 126-130).

Page 7. Line 161-163. Provide a reference for the Senegalese population used to compare the proportion of each age group recruited.

Authors’reply: The reference for the Senegalese population used for the proportion of each age group is provided in the revised manuscript (Please, see Line 172).

Page 8. Line 183-189. Information on individuals concerned with preparing the smear should be provided. Were they trained?

Authors’reply: Individuals involved in the preparation of the smear were trained on the sample collection and the preparation of the smears. The manuscript is revised to reflect this information Please, see Line 201-204).

Page 12. Table 1. The table shows that 43.2% had no occupation, while 25.7% were students. What, then, is the main source of livelihood for most of the populace?

Authors’reply: In the study area, the main source of livelihood for most of the population is farming and gold mining^[1]^. This figure is well represented among adults in the study population, with around 60% (farming 21.2% and gold mining 38.4%) (Please, see Table 1).

Table1. page 12. Different spellings were used for the “Quranic” school….”Koranic”….”Choranic”. Kindly reconcile the spelling.

Authors’reply: The manuscript is revised and “Koranic school” is used throughout the document.

Page 14-15. Consistent use of abbreviation for Plasmodium falciparum as P. falciparum. Ensure the same abbreviation after the initial full written form.

Authors’reply: The manuscript is revised in accordance. P. falciparum has been used throughout the manuscript as abbreviation for Plasmodium falciparum.

4. Discussion.

Page 22. Line 452-458. The study limitation, which considers the microscopy method used in this community study to be a possibility of missed diagnosis in low-density infection, may need to be revised.

In a study by Berzosa et al. 2018 in Equatorial Guinea, the microscopy used in your research is still recommended by WHO as it is cheap, allows for species differentiation, and is the acclaimed gold standard for diagnosis by WHO.

“Berzosa, P., de Lucio, A., Romay-Barja, M. et al. Comparison of three diagnostic methods (microscopy, RDT, and PCR) for detecting malaria parasites in representative samples from Equatorial Guinea. Malar J 17, 333 (2018). https://doi.org/10.1186/s12936-018-2481-4” three metho

Authors’reply: The authors would like to thank the reviewer for this comment. This limitation has been removed from the revised manuscript (Please, see Line 512-513).

1. Agence nationale de la statistique et de la démographie (ANSD), 2021.
